# Development of dendritic cell loaded MAGE-A2 long peptide; a potential target for tumor-specific T cell-mediated prostate cancer immunotherapy

**DOI:** 10.1186/s12935-023-03108-0

**Published:** 2023-11-11

**Authors:** Parisa Bakhshi, Maryam Nourizadeh, Laleh Sharifi, Mohammad M. Farajollahi, Monireh Mohsenzadegan

**Affiliations:** 1 Department of Medical Biotechnology, School of Allied Medical Sciences, Iran University of Medical Sciences (IUMS), Hemmat Highway, Tehran, Iran; 2 Immunology, Asthma and Allergy Research Institute, Tehran University of Medical Sciences, Tehran, Iran; 3 Uro-Oncology Research Center, Tehran University of Medical Sciences, Tehran, Iran; 4 Department of Medical Laboratory Sciences, School of Allied Medical Sciences, Iran University of Medical Sciences (IUMS), Hemmat Highway, Tehran, Iran

**Keywords:** Dendritic cell vaccine, Immunotherapy, MAGE-A2, Long peptide, Prostate cancer

## Abstract

**Background:**

Prostate cancer (PCa) is the second leading cause of cancer-related deaths among men worldwide. Immunotherapy is an emerging treatment modality for cancers that harnesses the immune system’s ability to eliminate tumor cells. In particular, dendritic cell (DC) vaccines, have demonstrated promise in eliciting a tumor-specific immune response. In this study, we investigated the potential of using DCs loaded with the MAGE-A2 long peptide to activate T cell cytotoxicity toward PCa cell lines.

**Methods:**

Here, we generated DCs from monocytes and thoroughly characterized their phenotypic and functional properties. Then, DCs were pulsed with MAGE-A2 long peptide (LP) as an antigen source, and monitored for their transition from immature to mature DCs by assessing the expression levels of several costimulatory and maturation molecules like CD14, HLA-DR, CD40, CD11c, CD80, CD83, CD86, and CCR7. Furthermore, the ability of MAGE-A2 -LP pulsed DCs to stimulate T cell proliferation in a mixed lymphocyte reaction (MLR) setting and induction of cytotoxic T cells (CTLs) in coculture with autologous T cells were examined. Finally, CTLs were evaluated for their capacity to produce interferon-gamma (IFN-γ) and kill PCa cell lines (PC3 and LNCaP).

**Results:**

The results demonstrated that the antigen-pulsed DCs exhibited a strong ability to stimulate the expansion of T cells. Moreover, the induced CTLs displayed substantial cytotoxicity against the target cells and exhibited increased IFN-γ production during activation compared to the controls.

**Conclusions:**

Overall, this innovative approach proved efficacious in targeting PCa cell lines, showcasing its potential as a foundation for the development and improved PCa cancer immunotherapy.

**Supplementary Information:**

The online version contains supplementary material available at 10.1186/s12935-023-03108-0.

## Background

Prostate cancer (PCa) remains the second leading cause of cancer-related deaths in men around the world. Despite the therapeutic benefit of general treatments, additional treatment strategies are needed to prevent progression from localized to advanced disease and to further improve survival outcomes for patients with advanced PCa [1]. Immunotherapy is a notable advancement in cancer treatment, that aims to harness the immune system to identify tumor antigens and eliminate tumors while leaving normal tissues undamaged [2]. Dendritic cells (DCs), as professional antigen-presenting cells (APCs), have become a promising immunotherapeutic approach for the treatment of cancers. To generate an immune response against cancer cells, the patient’s own cells are isolated, activated, and then transferred autologously to the patient after being loaded with tumor antigens [3]. Among a plethora of tumor antigens, melanoma antigen gene (MAGE)-A was the first human tumor-associated antigen to be discovered at the molecular level [4]. It belongs to the larger family of cancer/testis antigens (CTAs), whose expression is found in cancers as well as germinal cells [5]. They have been identified in a variety of cancers through different histological origins including myeloma [6], triple-negative breast cancers [7], non-small cell lung cancers (NSCLC) [8], and PCa [9]. One of the CTAs overexpressed in different histological types of tumors is MAGE-A2 [10]. It is a strong p53 (tumor-suppressor transcription factor) inhibitor through histone deacetylase (HDAC)3 recruitment, and its expression decreases cellular senescence and increases proliferation [11]. In PCa, MAGE-A2 is the most highly upregulated CTA (> 800-fold) that causes the proliferation of cancer cells and decreases chemosensitivity [12]; therefore, it may serve as a perfect antigen that potentially could be used for PCa immunotherapy. In addition to the selection of an optimal antigen, the ideal peptide length has become a topic of discussion. One approach for enhancing the efficacy of DC vaccination is to use long peptide (LP) as the source of tumor antigens [13]. LPs stimulate both CD4 + and CD8 + T cell responses and have prolonged antigen presentation compared to short peptides [14, 15]. After internalization, a part of the LPs is degraded by the endosomal pathway, loaded onto MHC-II molecules, and subsequently identified by CD4^+^ T helper cells. The other parts are cross-presented by MHC-I molecules to activate CD8^+^ T cells through the cytoplasmic or vacuolar pathway [16]. Moreover, LPs are able to alleviate potential immune tolerance and enhance vaccine potency [17]. While, shorter peptides are frequently constrained by human leukocyte antigen (HLA) types, LPs can cover a broader spectrum of HLAs containing multiple epitopes to improve immunogenicity [18]. Therefore, LPs have more potential to induce sustained and effective antitumor activity responses. As the first step towards developing an effective DC vaccine, we searched for optimal antigenic MAGE-A2-LP as a stimulator of both CD4^+^ and CD8^+^ cells using online bioinformatic tools. We speculated that selected MAGE-A2-LP might be a valuable immunogenic source for DC pulsing and a particular therapeutic target. To the best of our knowledge, this is the first report investigating the potency of DCs loaded with MAGE-A2-LP in the context of antitumor immune responses for PCa. Here, we explored whether DCs loaded with MAGE-A2 LP could promote in vitro stimulation of T lymphocytes against prostatic cancer cell lines. Our preliminary results confirmed that MAGE-A2-LP loaded DCs could stimulate T cell proliferation and activate the cytotoxicity of T cells against selected PCa cell lines.

## Methods

### Patient characteristics and sample collection

All experimental procedures were approved by the Ethics Committee of the Iran University of Medical Sciences (IR. IUMS.1398.090). Informed consent was obtained from all participants prior to blood sample collection. PCa blood samples (n = 10) were collected from the Uro-Oncology Research Center of the Imam Khomeini Hospital Comlex- Tehran. Pathological reports were evaluated to obtain the diagnosis of PCa and Gleason grade patients. None of the patients received radiotherapy or hormonal therapy prior to diagnosis. The mean age of the patients was 64.20 years and they were diagnosed with high-grade PCa with a Gleason score ≥ 8. Table 1 summarizes the patient characteristics, including age, serum PSA levels, Gleason scores, and tumor stage (TNM) classifications.

**Table 1:** Patient characteristics

Patient ID	Age (years)	Serum PSA (ng/ml)	Gleason score	Tumor stage (TNM)
P1	67	53.7	9	T_3b_N_1_M_0_
P2	56	183.8	9	T_3b_N_1_M_1_
P3	63	27.3	8	T_3b_N_1_M_0_
P4	52	58	8	T_3b_N_1_M_0_
P5	74	896	9	T_3b_N_1_M_1_
P6	58	239.7	9	T_3b_N_1_M_0_
P7	81	69.1	8	T_3b_N_1_M_1_
P8	53	104	9	T_3b_N_1_M_1_
P9	66	38.8	8	T_3b_N_1_M_0_
P10	73	127.4	8	T_3b_N_1_M_1_

*PSA* prostate specific antigen, *TNM* tumor-node-metastasis

### Design and synthesis of MAGE-A2- LP

The MAGE-A2 protein sequence was retrieved from the National Center for Biotechnology Information (NCBI) database. To design MAGE-A2-LP, online bioinformatic tools were utilized. First, we analyzed the protein sequence for the presence of MHC class I and II binding regions using the online database Immune Epitope Database (IEDB). The MHC class I and II binding regions were identified in the protein sequence. The 30 amino acids MAGE-A2-LP (KTGLLIIVLAIIAIEGDCAPEEKIWEELSM) which contains the MHC class I and II binding regions was selected. Additional online bioinformatic tools such as: Rankpep, NETMHC2PAN, and EPIMHC were used to analyze the selected LP. Moreover, to explore homology between our chosen LP and other members of the protein family, MAGE-A2-LP was subjected to BLAST analysis using the NCBI database. This approach enabled us to identify sequences in the database that bear a close resemblance to our LP, thereby providing valuable insights into its homology or similarity to established proteins. The results exhibited notable homology between selected LP and other members of the MAGE family including MAGE-A12, MAGE-A3, and MAGE-A6. The designed MAGE-A2-LP was synthesized by the Biomatik Corporation (Canada) and the purity of the peptide was determined by high-performance liquid chromatography (HPLC) and mass spectrometry analysis (Additional files 1 and 2, respectively). The freeze-dried MAGE-A2-LP was received, diluted in sterile water at 2 mg/ml and stored at − 20 °C for future use.

### Generation of monocyte-derived dendritic cells

Forty milliliter blood sample was collected from patients diagnosed with PCa using K3-EDTA tubes. Monocyte-derived dendritic cells (Mo-DCs) were generated using standard protocols. Peripheral blood mononuclear cells (PBMCs) were isolated using Ficoll-Hypaque gradient centrifugation [18]. Monocytes were purified from PBMCs through a negative selection protocol using the Pan Monocyte Isolation Kit (Miltenyi Biotec, Germany). For this purpose, PBMCs were labeled with the cocktail of biotin-conjugated monoclonal antibodies against antigens that were not expressed on human monocytes and then incubated with anti-biotin MicroBeads to firstly isolate the unbound monocytes through a magnetic-activated cell separation (MACS) column (Miltenyi Biotec, Germany). After removing the column from the magnet and firmly pushing the plunger into the column, the magnetically labeled nonmonocytes were flushed out and stored at − 80 °C as the T cells intended for coculturing with DCs in subsequent experiments. The number of collected monocytes and their viability were assessed by trypan blue staining. To obtain immature DC (imDCs), monocytes (1 × 10^6^ cells/mL) were cultured in RPMI-1640 complete medium (Invitrogen, USA) supplemented with 10% FBS (Gibco, Germany), 100 U/mL penicillin-streptomycin (Biowest, France), 100 ng/mL recombinant human GM-CSF (Biolegend, USA), and 100 ng/mL recombinant human IL-4 (Biolegend, USA) in a humidified incubator at 37 °C with 5% CO_2_ for five days. Every 3 days, half of the medium was refreshed with the same concentration of GM-CSF and IL-4. To pulse the DCs with MAGE-A2-LP, cultures were exposed to 10 µg/ml peptide for 4 h [19]. Then, they were matured by adding 2 µg/ml lipopolysaccharide (LPS, Sigma-Aldrich, USA) for 48 h [20].

### Immunophenotyping characterization of DCs by flow cytometry

To verify the differentiation and maturation of cells, flow cytometry analysis was performed using fluorochrome-labeled monoclonal antibodies (mAbs): FITC mouse anti-human CD14, CD40, CD11c and CD80, PE mouse anti-human HLA-DR and CD86, and APC mouse anti-human CCR7 and CD83, purchased from BioLegend, USA on an Attune NxT flow cytometer from Thermo Fisher Scientific, USA. Monocytes, imDCs, and mature DCs (mDCs) were collected on days 0, 5, and 7 of the culture periods using cell scrapers and pipettes. The collected cells were then centrifuged, resuspended in ice-cold FACS buffer, and aliquoted in FACS tubes followed by addition of conjugated mAbs. The tubes were incubated on ice in the dark for 45 min, washed, and resuspended in the FACS buffer for immediate analysis. For the isotype control, mouse immunoglobulin (IgG) was used at the same concentration as the other antibodies. FlowJo software (version. 10) was used for data analysis.

### T cell proliferation assay by mixed lymphocyte reaction

The stimulatory potential of DCs (imDCs and LP-loaded mDCs) was evaluated in mixed leukocyte reaction (MLR) condition and compared with control wells containing T cells alone (negative control) and T cells stimulated with IL-2 (positive control). CD14-negative cells obtained from LS column flushing (Miltenyi Biotec, Germany) post monocyte isolation underwent two phosphate buffered saline (PBS) washes and preserved in 10% dimethyl sulfoxide (DMSO, Sigma-Aldrich, USA) with FBS at − 80 °C for 24 h and then transferred to liquid nitrogen until they were needed for further experiments. The nonadherent portion of thawed CD14-negative cells that cultured for two hours were considered T cells. T cells were stained with carboxyfluorescein diacetate succinimidyl ester (CFSE, CFSE cell division tracker kit, BioLegend, USA) according to the manufacturer’s instructions. CFSE-labeled T cells (5 × 10^5^ cells) were seeded in a 96-well plate and cocultured with immature and LP-loaded mDCs at a ratio of 10:1 (T: DC) in the presence of 10 ng/mL IL-2 (Biolegend, USA) for 5 days at 37 °C in 5% CO2. After the 5-day coculture, T cells were washed in cold PBS. The proliferation rate was assessed using flow cytometry. In this regard, by FlowJo software (version. 10) the division index was calculated as the average number of cell divisions in the original population. The diameter of each colony generated after being cocultured was measured by Fiji (ImageJ software, NIH, USA) on inverted microscopic pictures. More than 10 independent microscopic fields were counted for each group.

### MAGE-A2 expression in PC3 and LNCaP prostate cancer cell lines

Two PCa cell lines, PC3 and LNCaP purchased from the Pasteur Institute of Iran, were selected as the target cells for conducting the cytotoxicity assay. To this end, the expression of MAGE-A2 in both cell lines was analyzed using flow cytometry. Isolated PBMCs from healthy donors were utilized as a control sample. The cells were seeded at a density of 1 × 10^5^ cells per well in a 24-well plate in RPMI supplemented with 10% (v/v) FBS and grown at 37 °C in a 5% CO2 for 24 h. Subsequently, the cells were fixed in 2% cold and freshly prepared paraformaldehyde in PBS and then incubated at room temperature for 15 min at slow shaking rate. To remove any extra fixative reagents, cells were washed with PBS and then centrifuged at 500×g for 5 min. For cell permeabilization, 200 µl of 0.5% Tween-20 (Sigma-Aldrich, USA) was added to each tube and incubated for 30 min. The samples were washed with PBS to remove Tween-20 from the medium. MAGE-A2 antibody (Biorbyt, UK) as primary antibody and FITC anti-rabbit IgG (Sigma-Aldrich, USA) as the secondary antibody were utilized to evaluate the MAGE-A2 expression in both cell lines by flow cytometry analysis.

### In vitro induction of antigen-specific CTLs

To activate CTLs, isolated autologous T cells were cocultured with LP-loaded mDCs at a ratio of 10:1, and they were restimulated twice with the same DCs on day 7 and day 14 and fed every 3 days with fresh medium containing IL-2. On day 21, the treated T cells were harvested, washed, and their cytotoxicity was analyzed.

### In vitro cytotoxicity assay

To test the ability of CTLs to kill target cells, they were cocultured with the PC3 and LNCaP cell lines as target cells. Each cell line was stained with CFSE to determine viability. The CTL assay was conducted at effector/target ratios of 0:1(target only as baseline control) [21], 10:1(unstimulated CTLs and mDC stimulated CTLs as effectors) and 20:1(mDC stimulated CTLs as effector) in 96-well round-bottomed culture plates, with a total volume of 200 µl, in triplicate. In addition, autologous PBMCs were used as a surrogate for normal cells to evaluate the safety of MAGE-A2-LP stimulated CTLs for normal cells. After 4 h of incubation at 37 °C in 5% CO2, flow cytometry was used to analyze the cytotoxic effect of CTLs against the PCa cell lines. To differentiate between live and dead cells, 10 µl of 0.5 mg/ml propidium iodide (PI) (final concentration of 25 µg/ml) was added to each sample immediately before reading by flow cytometer. The results were expressed as the percentage of CFSE + PI+ (double positive) cells.

### Cytokine detection by ELISA

On day 15, supernatants were separated from the coculture of imDCs and LP-loaded mDCs with autologous T cells as positive controls as well as T cells alone as negative controls and stored at − 20 °C until they were used for assessing interferon-gamma (IFN-γ). The level of IFN-γ secretion following activation and expansion of T cells was measured by an ELISA kit (IBL International, Germany) according to the manufacturer’s instructions, using a detection range of 1.6–100 pg/ml.

### Transwell migration assay

Transwell migration assay was performed using transwell chambers (8 μm pore size, SPL, South Korea). PC3 and LNCaP cells (2 × 10^4^ in each well), were cocultured with unstimulated CTLs and mDC stimulated CTLs at effector/target ratio of 10:1 along with PC3 and LNCaP cells alone as respective controls. Cells were seeded in the upper chambers of the 12 well plate in 500 µl serum-free medium. The lower chambers were filled with 1 ml complete medium. The chamber was incubated at 37 °C for 48 h. At the end of incubation, the cells in the upper surface of the membrane were removed with a cotton swab. Cells in lower chamber were fixed with methanol and stained with Giemsa. The images were taken with inverted microscope and analyzed using Fiji (ImageJ software, NIH, USA).

### Cell invasion assay

The matrigel invasion assay and its subsequent analyses were done as the procedure described in the transwell migration assay section. However, it should be noted that in the context of the cell invasion assay, transwell chambers were coated with 1 mg/ml matrigel (Corning, USA) prior to the assay.

### Statistical analysis

Each experiment was performed at least three times and data are reported as the mean values ± SD. Student’s t *test* was used to compare two groups with a parametric distribution. *P* values less than 0.05 were considered significant and values less than 0.01, 0.001, and 0.0001 are shown in the figures using **, *** and ****, respectively. All statistical analyses were carried out using GraphPad Prism version 8.0 for Windows (GraphPad Software, La Jolla, USA).

## Results

### Successful differentiation of immature and LP-loaded mature DCs from patients’ isolated monocytes

**Fig. 1:**
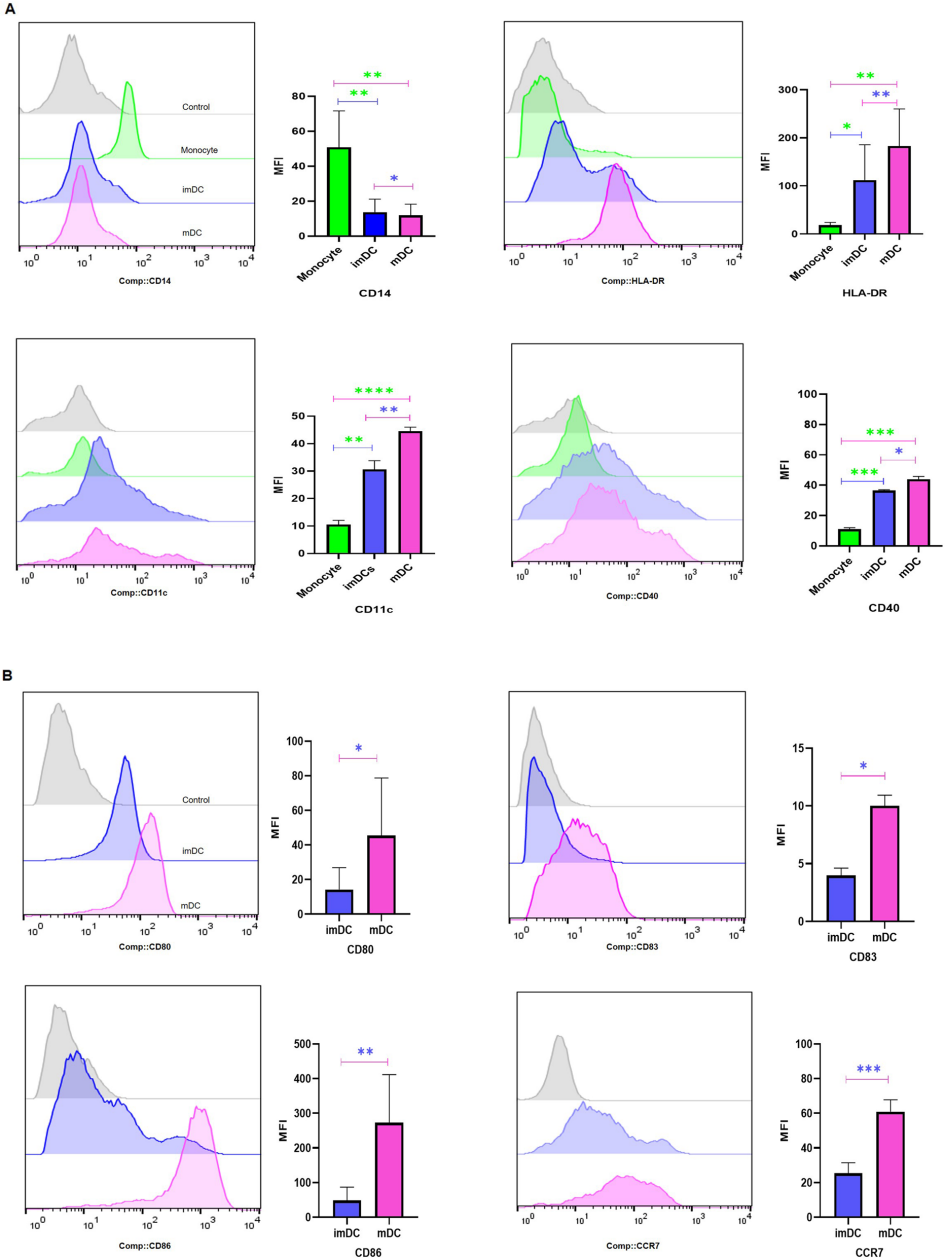
Flow cytometric and quantitative bar chart analyses of surface marker expression. **A** Expression of CD14, HLA-DR, CD11c, and CD40 are shown among monocytes, imDCs, and mDCs (n = 10). **B** Expression of CD80, CD83, CD86, and CCR7 are shown between imDCs and mDCs (n = 10). Histograms show the expression of each marker in a representative experiment, and the bar diagrams indicate the mean MFI ± SD for each marker. All groups showed significant differences in the expression of surface markers. A paired *t* test was used to compare the differences between two groups. *p ≤ 0.05, **p ≤ 0.01, ***p ≤ 0.001, ****p ≤ 0.0001. Control: isotype control, imDC: immature DCs, mDC: LP-loaded mature DCs

An LS-MACS column was used to isolate unbound monocytes that became imDCs after 5 days of exposure to the DC-generating cytokine cocktail including IL-4 and GM-SCF. The MAGE-A2-LP-pulsed imDCs were matured with the addition of LPS for 2 more days. Flow cytometry was used to determine whether CD14, HLA-DR, CD11c, and CD40 surface markers were differently expressed in monocytes, imDCs, and LP-loaded mDCs. As shown in Fig. 1A, monocytes expressed significantly more CD14 in comparison with imDCs and mDCs samples (p = 0.003, and p = 0.002, respectively). In contrast to CD14, HLA-DR, CD11c, and CD40 were significantly expressed at lower levels in monocyte samples and showed increased pattern in imDCs (p = 0.04, p = 0.002, and p = 0.0002, respectively) and mDCs (p = 0.008, p ≤ 0.0001, and 0.0003, respectively). Flow cytometry analysis was used to compare the expression level of each surface marker including CD80, CD83, CD86, and CCR7 among imDC and mDC samples. LP-loaded mDCs displayed high percentages of expression levels of CD80 (p = 0.02), CD83 (p = 0.01), CD86 (p = 0.004), and CCR7 (p = 0.0005) which were significant for all markers in comparison with their immature counterparts (Fig. 1B).

### LP-loaded matured DCs can effectively stimulate T cell responses

**Fig. 2:**
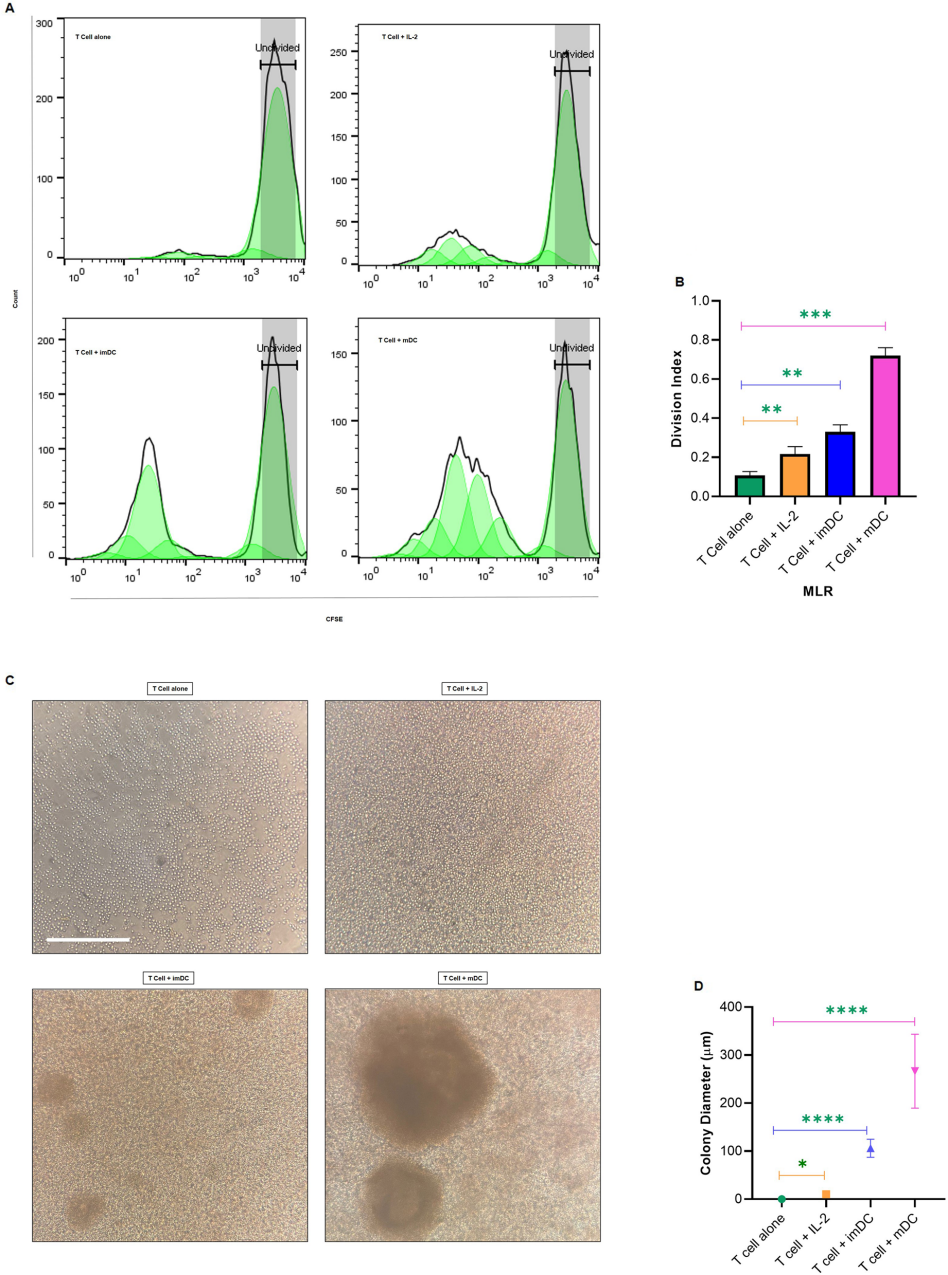
The proliferation of T cells cocultured with DCs. **A** Flow cytometry histogram of CFSE dilution from one representative experiment are shown. CFSE-labeled T cells were stimulated by IL-2, immature DCs and LP-loaded mature DCs and without stimulation as a control after 5 days. The proliferation of CFSE-labeled T cells was determined using flow cytometry. **B** Quantitative data from the division index (average division numbers that a cell in the original population had gone through; the average also takes into account the undivided cells) for each group from four independent experiments showing significance between different groups and a higher percentage for LP-loaded mDCs. **C** Colonies generated by each group of experiments. Bar: 200 μm and applied to all images. **D** Size comparison of generated colonies indicated a significant increase in colony diameter from LP-loaded mDCs compared with the other groups. All groups showed significant differences. A Paired *t* test was used to compare the differences between two groups. Data are presented as mean ± SD. *p ≤ 0.05, **p ≤ 0.01, ***p ≤ 0.001, ****p ≤ 0.0001. imDC: immature *DCs,* mDCs: LP-loaded mature DCs

The stimulatory capacity of DCs was assessed by coculturing of T cells with both imDCs (without pulsing) and mDCs (loaded with MAGE-A2-LP) for 5 days. The T cells were labeled with CFSE, and their proliferation was evaluated by CFSE dilution. IL-2 treated T cells served as the positive control. As it is shown in Fig. 2A and B, T cells treated with MAGE-A2 loaded mDCs displayed a significantly higher proliferation rate (p = 0.0007) than those treated with imDCs (p = 0.002) and IL-2 treated (p = 0.008). In addition, the colonies generated in the co-culture of T cells and LP-loaded mDCs were larger than those in other groups (Fig. 2C and D). Hence, mDCs loaded with MAGE-A2-LP had a remarkable effect on enhancing T cell growth.

### MAGE-A2-LP pulsed DCs activated T cells cytotoxicity

**Fig. 3:**
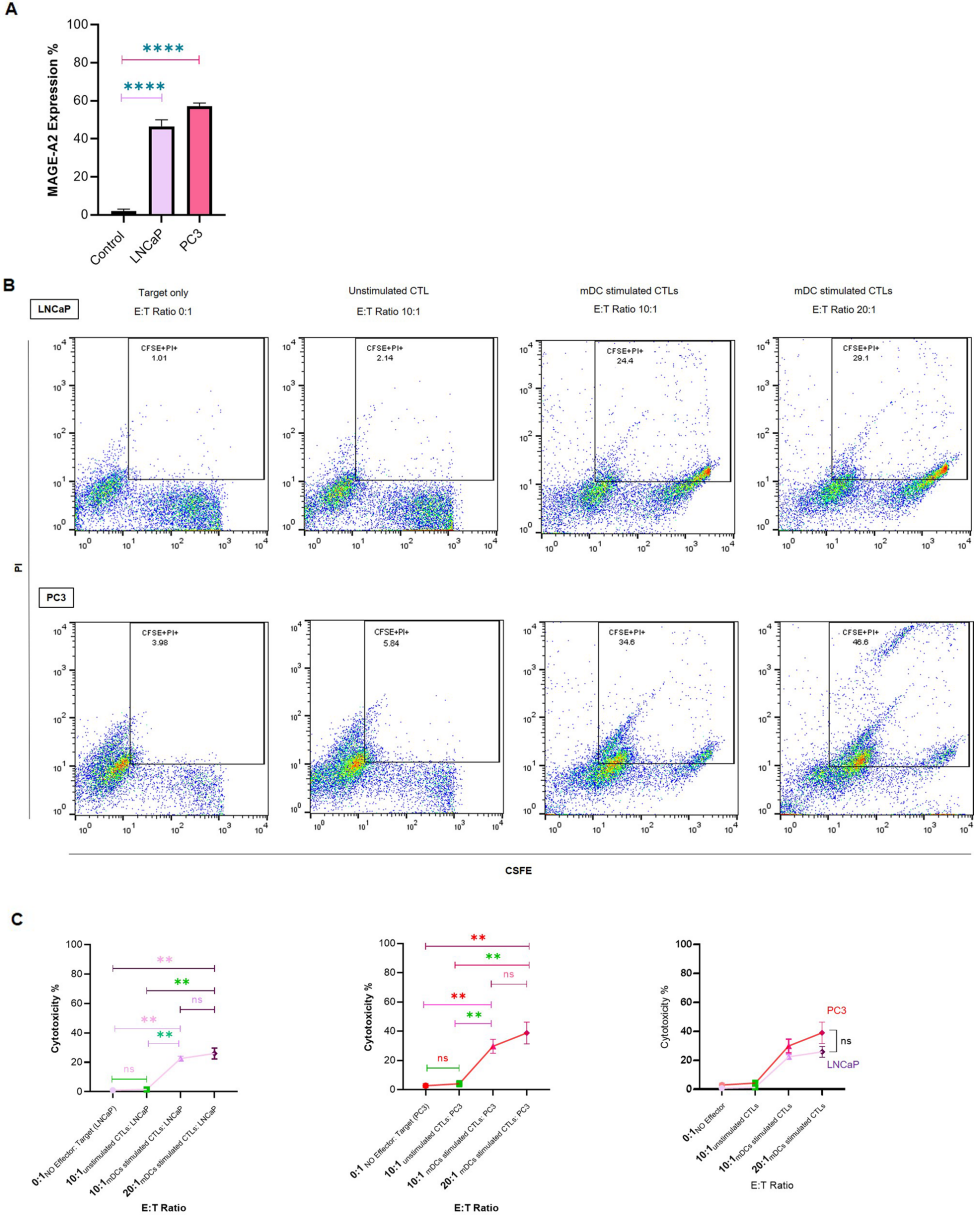
MAGE-A2 expression and cytotoxic activity against LNCaP and PC3 cell lines. **A** Quantitative analysis of the percentage of MAGE-A2 expression by flow cytometric assessments. **B** One representative dot plot of flow cytometry indicates the CFSE + PI + population. **C** Comparison of the percentage of cytotoxicity activity in 0:1(target only), 10:1(unstimulated and mDC stimulated CTLs) and 20:1(mDC stimulated CTLs) effector/target (E:T) ratios in LNCaP and PC3 cell lines as target cells. The data represent the mean ± SD. ns: not significant, **p ≤ 0.01, ****p ≤ 0.0001

To further monitor the functional consequence of DC-based T cell activity, we evaluated the cytotoxic activity of MAGE-A2-LP pulsed DC activated T cells against both PCa cell lines (PC3 and LNCaP). MAGE-A2 expression was assessed by flow cytometry in selected cell lines as well as in PBMCs as a control. Figure 3 A shows that both cell lines express MAGE-A2 as an antigen at significantly higher levels than the control sample (both p ≤ 0.0001). Therefore, these two cell lines were selected as targets for the cytotoxicity assay. Autologous T cells isolated from patients were cocultured with MAGE-A2-LP pulsed mDCs for 21 days at ratios of 10:1 and 20:1. After two stimulations with the same DCs and feeding with IL-2, the killing capacity of the resultant CTLs was assessed by CFSE-labeled PCa cell lines (PC3 and LNCaP). The rate of killing the target cells was determined based on the percentage of dead cells in the live cell population. Although the cytotoxicity levels in both PC3 and LNCaP in the ratios of 10:1 and 20:1 were not significantly different (p = 0.11; and p = 0.28, respectively), the highest cytotoxicity level was observed in PC3 cells at a 20:1 ratio (Fig. 3B and C). Furthermore, cytotoxicity of CTLs against normal cells (PBMCs) was conducted to evaluate the safety of MAGE-A2-LP stimulated CTLs for normal cells. The results of this experiment have been shown in Additional File 3. Notably, these results indicate that there were no significant differences in cytotoxicity of CTLs among normal cells without co-culturing, and co-culturing with different CTLs ratios, underscoring the safety and selectivity of our approach.

### T cells cocultured with MAGE-A2 pulsed DCs produced IFN-γ

**Fig. 4:**
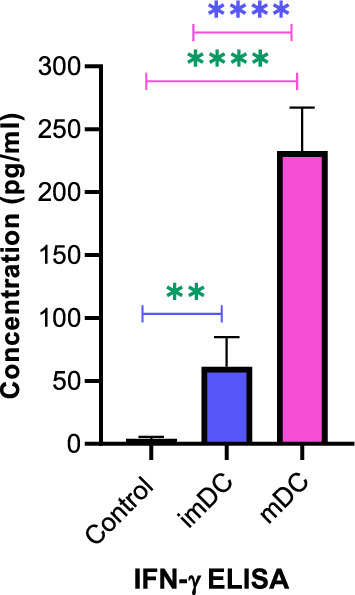
The concentration of IFN-γ. The IFN-γ concentration was analyzed by ELISA in the coculture supernatants of T cells stimulated with imDCs and mDCs. T- cells without coculturing were used as controls. A *t* test was used to compare the differences between two groups. Data are presented as the mean ± SD. **p ≤ 0.01, ****p ≤ 0.0001. imDC: immature DCs; mDC: LP-loaded mature DCs

MAGE-A2-LP pulsed DCs induced cytokine production in T cells in coculture. Mainly, mean IFN-γ production of T cells Cocultured with MAGE-A2 loaded DCs was 232.6 pg/ml which indicated a significantly higher concentration than the IFN-γ concentration in T cells stimulated by imDCs and control (T-cell without stimulation) samples (both p ≤ 0.0001) (Fig. 4).

### Stimulated CTLs with LP-loaded matured DCs can effectively decreased migration and invasion of PCa cell lines

**Fig. 5:**
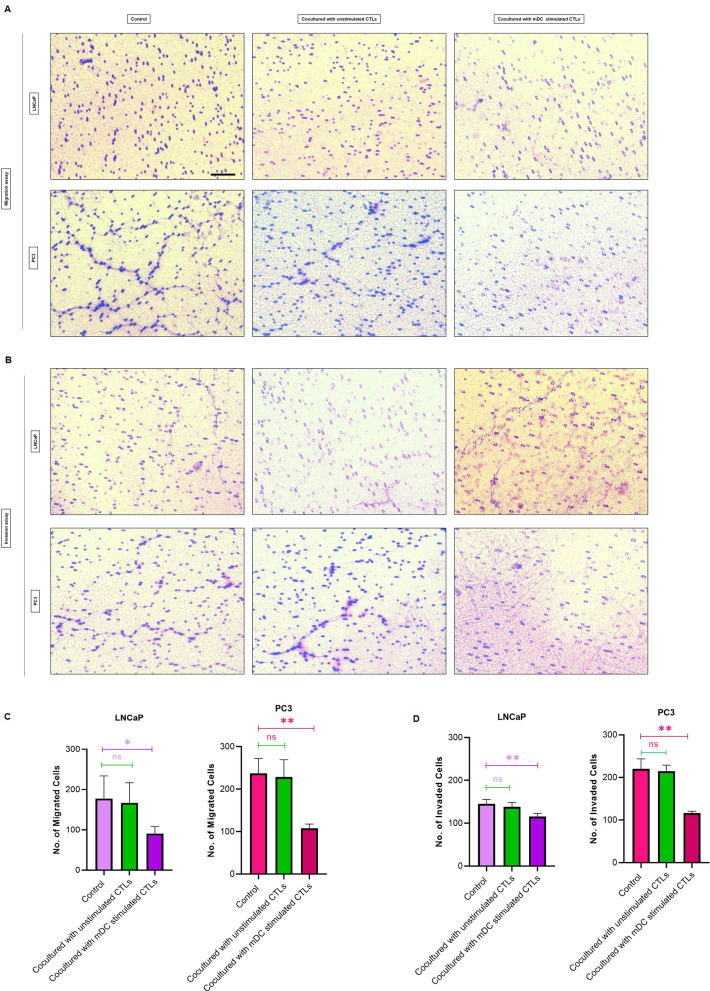
Transwell migration and invasion assays: **A** Representative images of transwell migration assay of coculturing LNCaP and PC3 cells with unstimulated CTLs, mDC stimulated CTLs, and respective control. **B** Representative images of matrigel invasion assay of coculturing LNCaP and PC3 cells with unstimulated CTLs, mDC stimulated CTLs, and respective control. **C** Quantitative graphical representations of transwell migration assay of LNCaP and PC3. **D** Quantitative graphical representations of invasion assay of LNCaP and PC3. The data represent the mean ± SD. ns: not significant, *p ≤ 0.05, **p ≤ 0.01

To investigate whether stimulated CTLs can affect cell motility and invasiveness of PCa cells, transwell cell migration and matrigel invasion assays were performed. we used unstimulated CTLs and mDC stimulated CTLs at ratio of 10:1 effector/target along with PC3 and LNCaP cells alone as controls (Fig. 5A and B). While unstimulated CTLs did not affect the motility and invasiveness of LNCaP and PC3 cells, coculturing mDC stimulated CTLs with LNCaP and PC3 significantly reduced cell migration (Fig. 5C). Similarly, coculturing mDC stimulated CTLs with LNCaP and PC3 significantly decreased the ability of cells to invade (Fig. 5D).

## Discussion

The oncogenic activity of MAGE-As as well as their immune-privileged nature, make them perfect antigens with the capacity to elicit highly specific immune responses for cancer [22]. The data from our experiment support the idea that MAGE-A2 could be a potential target antigen to induce tumor-specific cytotoxicity. We observed a substantial increase in the activation of T cell responses against MAGE-A2-expressing PCa cell lines. Numerous MAGE-A-based therapeutic cancer vaccines have been developed in recent years. The administration of recombinant MAGE-A3 protein in patients with lung cancer was one of the clinical studies conducted by using MAGE-A in cancer immunotherapy with promising initial data [23]. Based on our investigation, the BLAST analysis conducted on the selected MAGE-A2-LP displayed notable homology between the chosen LP and some members of the MAGE family, such as MAGE-A3, with 93% homology, indicating that MAGE-A2 could be a potential tumor antigen for cancer immunotherapy. In the context of PCa, Suyama et al., demonstrated that MAGE-A2 exhibited the highest level of upregulation among other CTAs. Moreover, upon the silencing of MAGE-A2 expression, a significant decrease in cancer cell proliferation and an increase in chemosensitivity were observed [12]. Therefore, MAGE-A2 might be a promising candidate for PCa immunotherapy.

One of the distinguishing aspects of our research lies in the use of LP as the antigen. Unlike short peptides, LPs encompass larger regions of the target protein sequence, which allows for the presentation of multiple epitopes and enhances the likelihood of eliciting a more diverse and potent immune response [24]. Several successful studies have utilized LP as a source of tumor antigens, as evidenced in subsequent literature citations. In line with our study, Bijker et al.; performed a comparison of different peptide vaccination strategies and showed superior performance of LPs over short peptides [17]. Furthermore, Patel et al., conducted a successful study to evaluate the safety and immunogenicity of LP; using seven different LPs, including MAGE-A1 and MAGE-A10 [25]. Ott et al.; synthesized up to 20 LPs to generate vaccine for melanoma patients. They observed that four of the six patients did not exhibit any subsequent recurrences after a period of 25 months post vaccination [26]. Collectively, it is plausible that LP could be a superior option for the development of a safe and efficacious DC vaccine.

The DC maturation status plays a pivotal role in the induction of immune responses following DC-based vaccination [27], where decreased expression of CD14 and increased expression of key costimulatory and maturation molecules (e.g. CD80, CD83, CD86 and HLA-DR) are involved in T cell activation [28]. Our results in DC phenotypic maturation status, indicated significant differences in the expression levels of CD14, HLA-DR, CD80, CD83, and CD86 in LP-loaded mDCs compared to the monocyte and immature groups. Moreover, the outcomes of our study demonstrated that CD86 was expressed in at higher levels in comparison to other costimulatory molecules. Previous studies conducted on CD80 and CD86 knockout mice have indicated that CD86 plays a significant role in the activation of T cells compared to CD80 [29]. In addition, another study suggested that CD86 may intensify DC:T cell interactions more prominently than CD80 [30]. Hence, the capability of CD86 to stimulate stronger DC:T cells plays a crucial role in initiating immune responses.

Full activation of T cells depends on the coordination of three distinct signals. In signal 1, DCs present peptide-MHC complexes to T cells for recognition by specific T cells, signal 2 involves the binding of costimulatory molecules, and signal 3 results from the cytokine environment regulating the proliferation and differentiation of T cells. For instance, IL-12 secreted by DCs promotes the T helper 1 (Th1) immune response [31]. Evaluating cytokine-12 has the potential to provide valuable insight into DC-T cell crosstalk [32]. However, we regret to report this, and it remains a limitation in our study.

In line with numerous studies that highlight the findings regarding a notable increase in T cell proliferation upon coculture with DCs loaded by tumor antigens in MLR assays and CTL responses [33, 34], our data demonstrated that utilizing MAGE-A2-LP pulsed DCs was effective for increasing T cell proliferation and killing PCa cell lines. Consistent with our data, Rabu et al.; provided evidence that LP could increase CD8-specific responses and inhibit the growth of transplanted tumors in mice. The data support the use of LPs to improve antitumor CD8^+^ T cell responses and therapeutic efficacy [35]. Multiple investigations have shown that IFN-γ helps effector responses of T cells and is considered to be a valuable indicator for detection of activation of antigen-specific T cells [32, 36, 37]. Here, we showed that tumor-specific T cells produced IFN-γ upon activation, and also LP-loaded mDCs were superior to imDCs in eliciting IFN-γ production from T cells.

Despite the promising results that make MAGE-LP a promising target antigen, several challenges need to be addressed to maximize the efficacy of T cell responses. To elicit the induction of effective T cell responses, it is important to consider the careful selection of optimal immunostimulators as well as the appropriate mode of antigen delivery to APCs [38]. Moreover, future studies should concentrate on identifying and optimizing antigen sequences that efficiently activate T cells against tumor antigens which can lead to enhanced immune recognition and response against cancer cells [13]. Furthermore, modification of the peptide structure as well as designing novel synthetic LPs that include CTLs and helper peptides could increase immunogenicity and improve clinical efficacy [39]. Considering clinical applications, DC vaccines show minimal side effects and low toxicity, which make them safe for patients; therefore, further investigations could prioritize integrating DC vaccines with additional therapeutic procedures such as combination therapy, immune checkpoint-targeting therapies, and personalized approaches to enhance treatment outcomes [40].

## Conclusions

Taken together, this is the first report on the use of MAGE-A2-LP to design a DC cancer vaccine, revealing that pulsed DCs generated from prostatic cancer monocytes were capable of inducing immune responses through T cell proliferation and cytotoxic activation. We suggest that MAGE-A2-LP pulsed DCs can be used in T cell mediated cancer immunotherapy for individuals with PCa. With this in mind, MAGE-A2-LP, as an appropriate candidate antigen in immunotherapy deserves further in vitro and in vivo investigations to better comprehend its potential as a weapon against PCa.

### Supplementary Information

**Additional file 1.** HPLC Analysis for synthesized MAGE-A2-LP purity.

** Additional file 2.** MS Analysis for synthesized MAGE-A2-LP purity. 

** Additional file 3.** Safety profile of cytotoxicity activity of MAGE-A2-LP CTLs co-culturing with PBMCs as normal cells.

## Data Availability

The datasets analyzed during the current study are available from the corresponding author on reasonable request.

## Abbreviations

PCa: Prostate cancer
DC: Dendritic cell
imDCs: Immature DC
mDCs: Mature DCs
LP: Long peptide
MLR: Mixed lymphocyte reaction
CTLs: Cytotoxic T cells
IFN-γ: Interferon-gamma
APCs: Antigen-presenting cells
MAGE: Melanoma antigen gene
CTAs: Cancer/testis antigens
NSCLC: Non-small cell lung cancers
HDAC: Histone deacetylase
HLA: Human leukocyte antigen
Mo-DCs: Monocyte-derived dendritic cells
PBMCs: Peripheral blood mononuclear cells
MACS: Magnetic-activated cell separation
LPS: Lipopolysaccharide
mAbs: Monoclonal antibodies
PBS: Phosphate-buffered saline
DMSO: Dimethyl sulfoxide
CFSE: Carboxyfluorescein diacetate succinimidyl ester
PI: Propidium iodide
Th1: T helper 1
