# Supplementary material for: Development of dendritic cell loaded MAGE-A2 long peptide; a potential target for tumor-specific T cell-mediated prostate cancer immunotherapy
Source: Cancer Cell Int. 2023 Nov 11;23:270. doi: 10.1186/s12935-023-03108-0 (PMC10638778; doi:10.1186/s12935-023-03108-0)
Supplement: Supplementary file 1 — Additional file 1. HPLC Analysis for synthesized MAGE-A2-LP purity. [file 12935_2023_3108_MOESM1_ESM.docx]

**Additional file 1**: HPLC Analysis for synthesized MAGE-A2-LP purity.

Column: 4.6×250mm, Sinochrom ODS-BP 5

Solvent A: 0.1% trifluoroacetic in 100% acetonitrile

Solvent B: 0.1% trifluoroacetic in 100% water

Gradient: A B

0.01min 30% 70%

25min 55% 45%

25.1min 100% 0%

30min STOP

Flow rate: 1.0 mL/min

Wavelength: 220nm

Volume: 5ul

Peak No. Ret Time Height Area Conc..

1 14.825 5439.236 48066.098 0.4539

2 15.273 42520.375 517038.594 4.8822

3 15.632 31833.461 257796.219 2.4343

4 15.853 608118.875 8498952.000 80.2522

5 16.138 60888.289 451069.531 4.2593

6 16.338 20479.521 185180.422 1.7486

7 16.538 18086.992 146996.125 1.3880

8 16.653 17907.584 171876.500 1.6230

9 17.002 3480.836 37481.051 0.3539

10 17.530 1259.932 13849.608 0.1308

11 19.903 9201.630 94665.578 0.8939

12 21.318 4332.682 33313.609 0.3146

13 23.345 13433.035 71822.813 0.6782

14 23.632 2696.582 21971.488 0.2075

15 24.577 4214.007 40222.363 0.3798

Total 100.0000
