# Supplementary figures and images for: Development of dendritic cell loaded MAGE-A2 long peptide; a potential target for tumor-specific T cell-mediated prostate cancer immunotherapy
Source: Cancer Cell Int. 2023 Nov 11;23:270. doi: 10.1186/s12935-023-03108-0 (PMC10638778; doi:10.1186/s12935-023-03108-0)

**Additional file 2**: MS Analysis for synthesized MAGE-A2-LP purity.


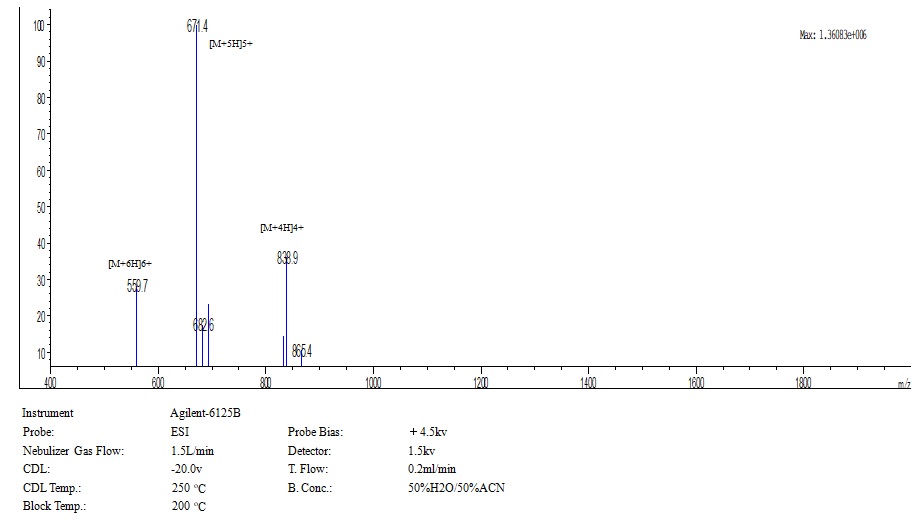

Supplement: Supplementary file 2 — Additional file 2. MS Analysis for synthesized MAGE-A2-LP purity. [file 12935_2023_3108_MOESM2_ESM.docx]
