# Supplementary material for: Development of dendritic cell loaded MAGE-A2 long peptide; a potential target for tumor-specific T cell-mediated prostate cancer immunotherapy
Source: Cancer Cell Int. 2023 Nov 11;23:270. doi: 10.1186/s12935-023-03108-0 (PMC10638778; doi:10.1186/s12935-023-03108-0)
Supplement: Supplementary file 3 — Additional file 3. Safety profile of cytotoxicity activity of MAGE-A2-LP CTLs co-culturing with PBMCs as normal cells. [file 12935_2023_3108_MOESM3_ESM.docx]

**Additional file 3:** Safety profile of cytotoxicity activity of MAGE-A2-LP CTLs co-culturing with PBMCs as normal cells.


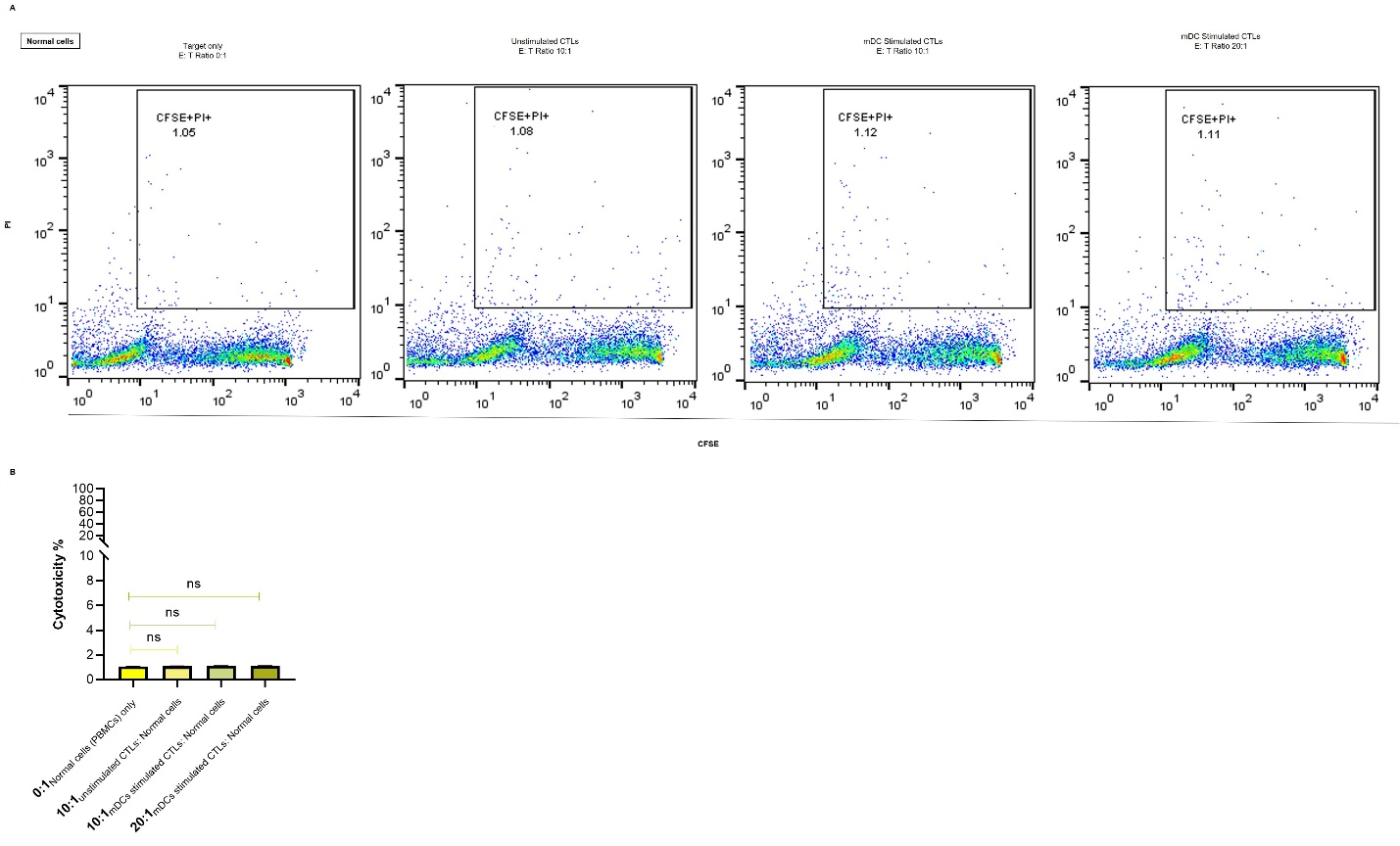


**Cytotoxic activity of MAGE-A2- LP stimulated CTLs against normal cells. A** One representative dot plot of flow cytometry indicates the CFSE+PI+ population. **B** Comparison of the percentage of cytotoxicity activity in 0:1(normal cells without co-culturing), 10:1(unstimulated and mDC stimulated CTLs) and 20:1(mDC stimulated CTLs) effector/target (E:T) ratios in PBMCs as normal cells. The data represent the mean ± SD. ns: not significant.
